# Microarray analysis of *Shigella flexneri*-infected epithelial cells identifies host factors important for apoptosis inhibition

**DOI:** 10.1186/1471-2164-11-272

**Published:** 2010-04-29

**Authors:** Christina S Faherty, D Scott Merrell, Cristina Semino-Mora, Andre Dubois, Aishwarya V Ramaswamy, Anthony T Maurelli

**Affiliations:** 1 Department of Microbiology and Immunology, F. Edward Hébert School of Medicine, Uniformed Services University of the Health Sciences, 4301 Jones Bridge Road, Bethesda, MD 20814, USA; 2 Laboratory of Gastrointestinal and Liver Studies, Department of Medicine, F. Edward Hébert School of Medicine, Uniformed Services University of the Health Sciences, 4301 Jones Bridge Road, Bethesda, MD 20814, USA; 3 Current address: Center for Vaccine Development, Department of Pediatrics, University of Maryland School of Medicine, Baltimore, MD 21201-1509 USA

## Abstract

**Background:**

*Shigella flexneri *inhibits apoptosis in infected epithelial cells. In order to understand the pro-survival effects induced by the bacteria, we utilized apoptosis-specific microarrays to analyze the changes in eukaryotic gene expression in both infected and uninfected cells in the presence and absence of staurosporine, a chemical inducer of the intrinsic pathway of apoptosis. The goal of this research was to identify host factors that contribute to apoptosis inhibition in infected cells.

**Results:**

The microarray analysis revealed distinct expression profiles in uninfected and infected cells, and these changes were altered in the presence of staurosporine. These profiles allowed us to make comparisons between the treatment groups. Compared to uninfected cells, *Shigella-*infected epithelial cells, both in the presence and absence of staurosporine, showed significant induced expression of *JUN*, several members of the inhibitor of apoptosis gene family, nuclear factor κB and related genes, genes involving tumor protein 53 and the retinoblastoma protein, and surprisingly, genes important for the inhibition of the extrinsic pathway of apoptosis. We confirmed the microarray results for a selection of genes using *in situ *hybridization analysis.

**Conclusion:**

Infection of epithelial cells with *S. flexneri *induces a pro-survival state in the cell that results in apoptosis inhibition in the presence and absence of staurosporine. The bacteria may target these host factors directly while some induced genes may represent downstream effects due to the presence of the bacteria. Our results indicate that the bacteria block apoptosis at multiple checkpoints along both pathways so that even if a cell fails to prevent apoptosis at an early step, *Shigella *will block apoptosis at the level of caspase-3. Apoptosis inhibition is most likely vital to the survival of the bacteria *in vivo*. Future characterization of these host factors is required to fully understand how *S. flexneri *inhibits apoptosis in epithelial cells.

## Background

*Shigella flexneri *is a Gram-negative, facultative intracellular organism, and the causative agent of bacillary dysentery. Infection with *Shigella *causes an intense acute inflammatory reaction that leads to the destruction of the colonic epithelium [1]. Clinical symptoms include watery diarrhea, severe abdominal pain, and bloody, mucoid stools. These symptoms of dysentery are due to the penetration of *Shigella *into colonic epithelial cells, which provide an intracellular environment for the bacteria to multiply and spread to adjacent cells [1]. Entry into epithelial cells is mediated by the Ipa proteins encoded on the 220-kb virulence plasmid. Secretion of these proteins is dependent on a type III secretion system (T3SS), which is encoded by 20 genes in the *mxi-spa *locus of the virulence plasmid. Additional T3SS effector proteins are secreted through the T3 needle when the bacteria are inside the cytoplasm of the host cell [1].

We previously demonstrated that *S. flexneri *inhibits apoptosis in epithelial cells [2]. Apoptosis, or programmed cell death, is a form of cell death that occurs without damage or lysis to neighboring cells [3]. The intrinsic pathway of apoptosis is induced by various stimuli that leads to cytochrome *c *release from the mitochondria and activation of the caspase cascade while the extrinsic pathway of apoptosis is induced by cytokine receptors of the tumor necrosis factor (TNF) family [3]. In the presence of staurosporine (STS), a chemical inducer of the intrinsic pathway of apoptosis, *S. flexneri *inhibits apoptosis by preventing the activation of caspase-3 despite the fact that both cytochrome *c *release from the mitochondria and caspase-9 activation occur [2]. Given these findings, we next wanted to determine the important cellular changes that occur in epithelial cells upon infection with *S. flexneri *and subsequent exposure to STS. Previous research analyzed changes in eukaryotic gene expression due to *S. flexneri *invasion using whole genome arrays [4]; however, analysis in the presence of an apoptosis inducer has not performed. Therefore, the goal of this paper was to identify the changes in apoptosis-specific genes due to *S. flexneri *invasion both in the presence and absence of STS. This analysis will not only enhance our understanding of how *S. flexneri *survives inside epithelial cells, but also allow us to fully understand the mechanisms of protection from apoptosis by identifying the host factors involved in this process. The microarray analysis revealed distinct expression profiles in uninfected and infected cells, and these changes were altered in the presence of staurosporine. Based on these profiles, we made several comparisons between the treatment groups. Compared to uninfected cells, we found numerous alterations in host factors, including the jun oncogene, inhibitor of apoptosis gene family members, nuclear factor κB (NF-κB), and genes involving tumor protein 53 and the retinoblastoma protein, all of which are important for the pro-survival state of the infected cell. These data indicate that upon infection, the bacteria utilize multiple checkpoints along both pathways to prevent apoptosis. If *Shigella *fails to inhibit apoptosis at an early step, the bacteria will block apoptosis at the level of caspase-3. This inhibition is vital for the bacteria to survive *in vivo*.

## Results and Discussion

The treatments for the microarray analysis were selected based on published observations that *Shigella*-infected HeLa cells do not undergo apoptosis in the presence of STS while uninfected HeLa cells undergo apoptosis in the same conditions [2]. The bacteria were also able to inhibit apoptosis in the colonic T84 cell line (data not shown). The temporal strategy and length of STS exposure times and infection were chosen to highlight key points in the apoptosis pathway. These key points include the activation of pro-apoptotic proteins preceding damage to the mitochondria (0.5 to 1 hour STS incubation), cytochrome *c *release from the mitochondria (2 hour STS incubation), and activation of caspase-3 before significant damage to the HeLa nuclei (2.75 hour STS incubation).

**Figure 1:**
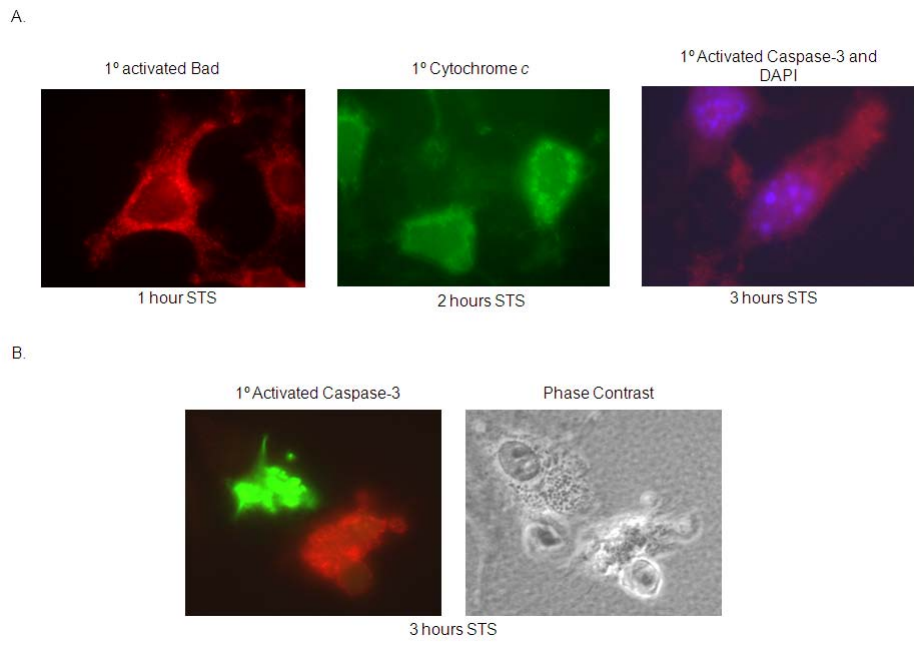
**Immunofluorescence analysis of HeLa cells treated with STS**. A. Uninfected cells stained with antibodies against key points in the STS-induced apoptosis pathway, namely Bad activation at 1 hour, cytochrome *c *release at 2 hours, and activated caspase-3 and DNA damage with DAPI nuclear staining at 3 hours STS treatment. B. A monolayer of HeLa cells was infected with *Shigella *harboring a low copy number plasmid expressing GFP to allow green fluorescence. Left: Merged image of the monolayer for GFP and the activated form of caspase-3. Only the uninfected cells have a positive signal for caspase-3 activation. Right: Phase contrast view to visualize the bacteria in the cytoplasm of the HeLa cell. Images are representative of three repeated experiments, and cell counts of at least 300 cells for each treatment demonstrated consistent results (data not shown).

To phenotypically verify that these incubation times mirrored the above expectations, we exposed uninfected HeLa cells to STS for 1, 2, or 3 hours and then performed immunofluorescence analysis (Figure 1A). After 1 hour of STS treatment, cells were stained with an antibody against the Bad protein to detect total levels of the protein (Figure 1A). Phosphorylation of Bad promotes binding to 14-3-3 proteins, which prevents the pro-apoptotic function of Bad [5]. An antibody that recognizes only the phosphorylated form of Bad yielded a weak signal (data not shown). Therefore, the pro-apoptotic Bad protein was active after 1 hour of STS treatment since the dephosphorylated form of Bad was primarily detected. Next, we tested for cytochrome *c *release from the mitochondria after 2 hours of STS treatment using a weak permeabilization treatment so only cytochrome *c *released from the mitochondria is stained and cytochrome *c *retained in the mitochondria produces only a weak signal [2]. The bright signal (Figure 1A) indicates that cytochrome *c *release occurred after the 2 hours of STS treatment. Finally, caspase-3 activation and nuclear damage was assessed after 3 hours of STS treatment using an antibody that recognizes only the activated form of caspase-3 and the DAPI nuclear stain, respectively, as previously described [2]. Caspase-3 activation with subsequent DNA damage was detected after 3 hours of STS treatment (Figure 1A). Control experiments verified that a 2.75 hour STS incubation time was adequate for caspase-3 activation without significant DNA damage (data not shown). In addition, to highlight our previous report that *S. flexneri *inhibits STS-induced apoptosis, we performed the apoptosis assay with a strain of bacteria expressing a green fluorescence protein (GFP) on a low copy plasmid. After the 6 hour assay, the infected cells were fixed and stained for activated caspase-3. As seen in Figure 1B, the uninfected cell is positive for activated caspase-3; whereas the adjacent infected cell only has a bright green signal due to the GFP expression by the bacteria and lacks a signal for activated caspase-3. Control experiments showed that the bacteria expressing GFP on the low copy plasmid had the same growth rate as wildtype bacteria (data not shown).

**Figure 2:**
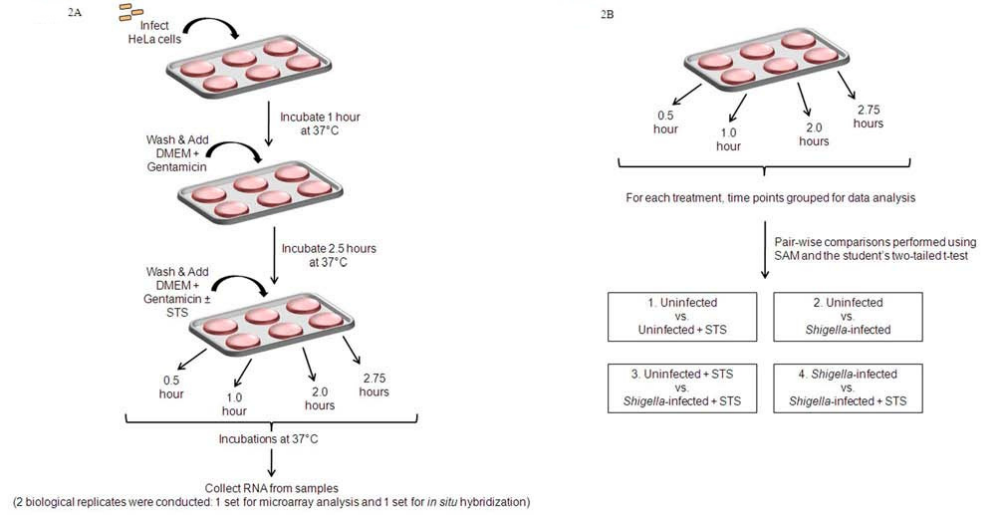
**Schematic of the infections and treatment conditions used in this study**. A. The infection and STS treatment conditions were modified to analyze a time course of STS exposure and adapted from the apoptosis assay previously described [2]. Briefly, *S. flexneri *was applied to a monolayer of HeLa cells and incubated at 37°C for 1 hour in Dulbecco's modified Eagle's medium (DMEM). Afterwards, the cells were washed and DMEM plus 50 μg/ml gentamicin was added to kill the extracellular bacteria and allow the intracellular bacteria to replicate. Uninfected cells received the same treatments and washes. Finally, infected and uninfected cells were washed and received DMEM plus gentamicin or DMEM plus gentamicin and 4 μM STS for 0.5, 1.0, 2.0, or 2.75 hours. After the treatments, the RNA was harvested from the cells. Two biological replicates were performed: one set for the microarray studies and one set for *in situ *hybridization (ISH) studies to confirm the microarray results. A single time point was analyzed for the ISH (see Methods). B. The individual time points were grouped together and then utilized as a single group in the pairwise comparisons for the microarray analysis. The four pairwise comparisons performed are indicated.

Based on these results, we chose to examine uninfected and infected cells in the presence and absence of STS using the strategy outlined in Figure 2. The 2.5-hour incubation of Dulbecco's modified Eagle's medium (DMEM) with 50 μg/ml gentamicin allows the intracellular population of bacteria to grow within the HeLa cells [2]. All STS treatments were matched with treatment controls in which no STS was added. After the treatments, the RNA was harvested at the indicated times, reverse transcribed, and prepared for hybridization as described in the Methods. The microarrays used are specific for apoptosis genes and contain approximately 20 000 spots representing 451 genes. The resulting microarray data were collated using the Stanford Microarray Database and utilized for pairwise analysis using the Statistical Analysis for Microarrays (SAM) algorithm and the student's two-tailed t-test to identify genes showing significant changes in expression.

**Figure 3:**
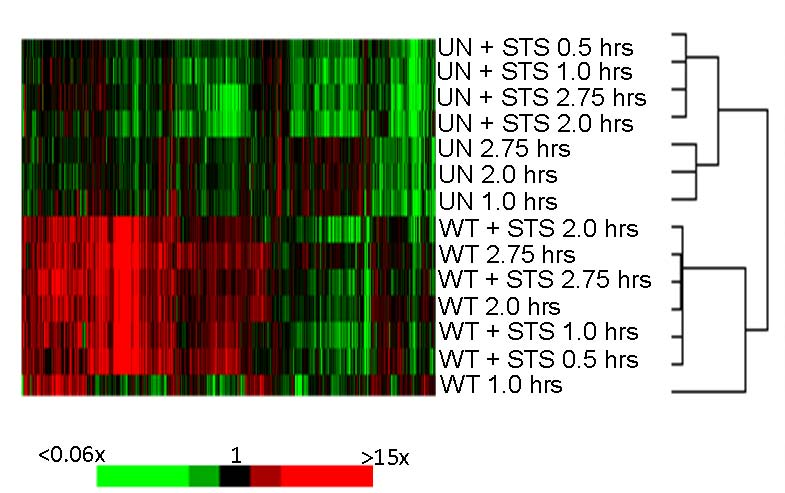
**Cluster diagram of significant genes across treatment groups**. Cluster diagram of the genes showing statistically significant differences in uninfected (UN) or wildtype-infected (WT) HeLa cells treated with or without staurosporine (STS) for the given time points. Red and green colors represent increased or decreased expression, respectively, relative to the reference sample. Note the dendogram, which displays the relationship of each treatment, shows two major nodes that segregate the majority of uninfected samples from wildtype-infected samples. The color scale represents the fold change from the reference for induced and repressed expression.

Additional file 1, Table S1 provides the data for all spots that showed statistically significant differences in the indicated pairwise analyses, and the complete data set is available at http://smd.stanford.edu. We performed four pairwise comparisons to identify key differences between the treatment groups, and in-depth discussion of these comparisons is provided below. The individual time points were grouped together and then utilized as a single group in the pairwise comparison. While this approach has the limitation of overlooking transient changes in host gene expression, we chose this method of analysis to identify the most consistent and significant changes since we reasoned that these changes would be vital for apoptosis inhibition during the entire six hours of infection. Additionally, due to cost limitations we chose to use one replicate of the experiment for microarray analysis and the other replicate for *in situ *hybridization (ISH) analysis. While this experimental design and the lack of a microarray replicate prevented statistical analysis using ANOVA, we were able to perform pairwise comparisons across variable pairs using SAM and the student's two-tailed t-test. While the analyses in the absence of a replicate are less than ideal, we compensated for this by performing extensive ISH analysis to confirm the most important changes detected in the microarray data. Additional file 2, Table S2 provides the list of genes in all comparisons categorized by function and provides brief descriptions of gene function obtained from NCBI's Entrez Gene. Cluster analysis of significantly changed genes across the treatment groups revealed that the arrays segregate into two major nodes (Figure 3). These major nodes cluster uninfected cells away from infected cells. Within the uninfected node, uninfected cells treated with STS segregate away from uninfected cells that did not receive STS treatment. However, this is not the case with infected samples; STS treated samples are interspersed with untreated samples across the node. This microarray analysis highlights clear differences in the expression of apoptotic genes in infected cells compared to uninfected cells, and interestingly, STS does not affect this pattern of apoptotic gene expression in infected cells. This analysis has provided insight into the strategies employed by *S. flexneri *to inhibit apoptosis in infected epithelial cells.

### Uninfected HeLa cells with STS compared to uninfected HeLa cells

There were 122 genes whose expression was significantly altered in uninfected cells treated with STS (USTS) versus uninfected cells (U). Interestingly, all 122 genes were repressed, which indicates that the cells receiving STS treatment turned off most gene expression during apoptotic death and suggests that the pro-apoptotic proteins already available in the cell are sufficient to induce death without *de novo *synthesis. These proteins include caspases, DNA repair enzymes, p53-associated genes, pro-apoptotic, and pro-survival genes (Additional file 2, Table S2).

### *Shigella*-infected HeLa cells compared to uninfected HeLa cells

**Figure 4:**
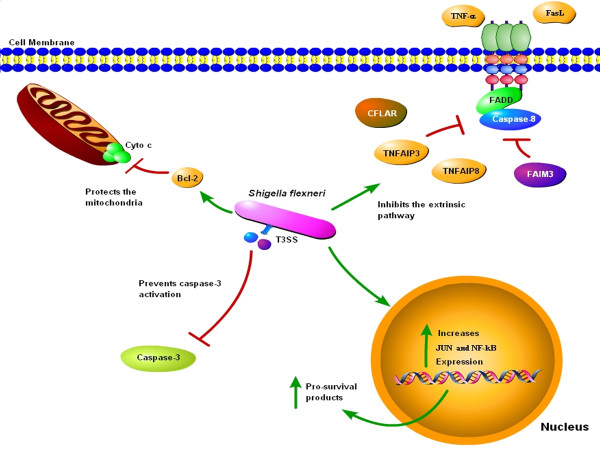
**Model of apoptosis inhibition by *Shigella flexneri *at multiple checkpoints in epithelial cells**. Infection of epithelial cells by *S. flexneri *results in several levels of protection from apoptosis. First, the bacteria prevent cytochrome *c *release from the mitochondria through the upregulation of BCL-2 proteins. Second, the extrinsic pathway of apoptosis is inhibited from *in vivo *stimuli such as TNF-α and FasL. This inhibition is most likely due to the upregulation of proteins like CFLAR, TNFAIP3, TNFAIP8, and FAIM3. Third, infection leads to the induced expression of JUN and NF-κB, which has many pro-survival effects including the increased expression of the IAPs (BIRC4, BIRC1, BIRC5, and BIRC7), the Bcl-2 family, and caspase-8 inhibitors. Finally, the bacteria prevent caspase-3 activation to provide downstream protection in the presence of strong apoptosis inducers. Through the use of T3SS effector proteins, the bacteria could directly generate mitochondrial protection, the inhibition of the extrinsic pathway, and caspase-3 inhibition. Alternatively, *S. flexneri *could indirectly produce these changes through the upregulation of pro-survival factors like JUN and NF-κB.

The analysis identified a total of 137 induced genes and 3 repressed genes in wildtype-infected HeLa cells (WT) compared to uninfected cells (U). In general, infected cells are in a pro-survival state compared to uninfected cells due to significant induction of various genes important for apoptosis inhibition (see Figure 4). First, there was a significant induction (17-fold) of *JUN *(also known as *c-JUN *or *AP-1*), which is a transcription factor and an oncogene [6]. The p38 MAPK and JNK signaling cascades induce JUN in response to pro-inflammatory cytokines and genotoxic stress. Upon activation, JNKs translocate to the nucleus to phosphorylate and enhance the transcriptional activity of JUN [7]. JUN has both pro-apoptotic and pro-survival gene targets, and it is hypothesized that the balance between these target genes is what determines whether the cell survives or undergoes apoptosis [7]. Lipopolysaccharides (LPS) are the major component of the outer membrane of Gram-negative bacteria, and have been shown to induce the expression of *JUN *[8,9]. It is therefore not surprising that the induction of *JUN *is so robust in WT cells. However, we cannot rule out the possibility that a bacterial T3SS effector protein expressed intracellularly also contributes to the induction of *JUN*. Similar induction was also seen in a previous microarray analysis of *Shigella*-infected cells [4]. Below we provide evidence that WT cells are in a pro-survival state, some of which may be due to *JUN *induction. JUN targets include cyclins, E2F transcription factors, Ras-GRF1 (a guanine-nucleotide exchange factor), and p53 [6,10-12]. Given that there is a significant increase in *JUN *expression, perhaps JUN is a major contributing factor to the pro-survival state of the infected cell. Future studies involving small interfering RNA (siRNA) to knock down *JUN *expression in infected cells will allow us to determine if *JUN *induction upon infection is essential for the pro-survival state of the cell. Moreover, we predict that *S. flexneri *mutants that are unable to inhibit apoptosis may not induce *JUN *to the extent seen in WT cells.

**Figure 5:**
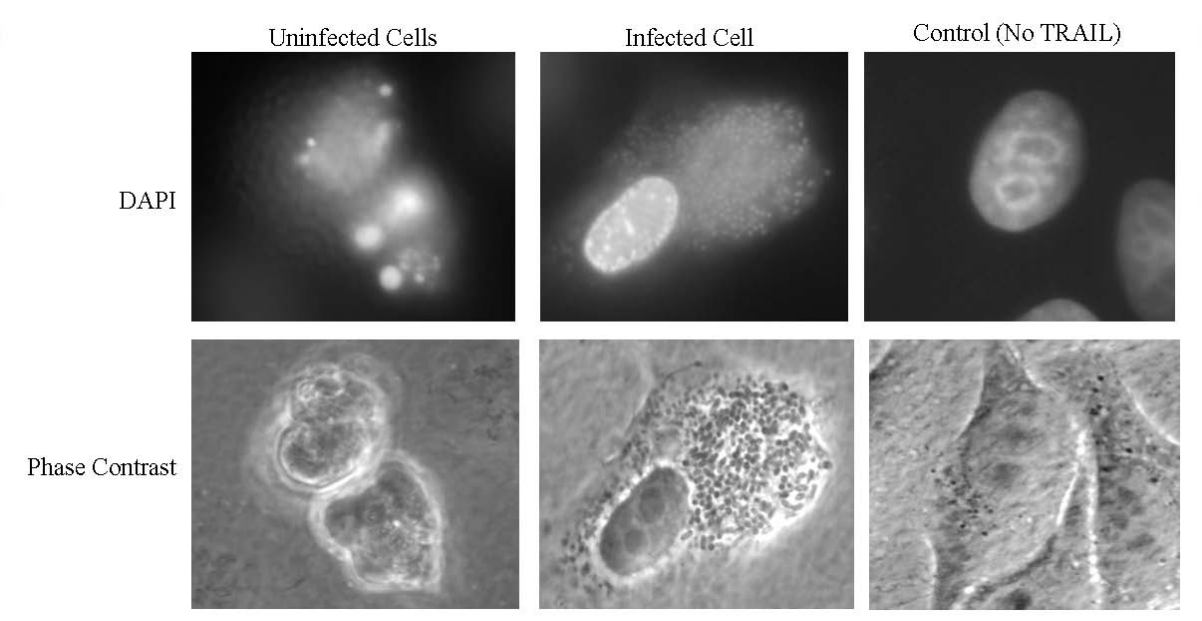
**Infected cells resist apoptosis induction via the extrinsic pathway**. Uninfected and wildtype-infected cells analyzed in the apoptosis assay treated with TRAIL. Top: DAPI stain of uninfected (left) and infected (center) cells treated with TRAIL. Note the uninfected cells have characteristic apoptotic nuclei while the infected cell, in which the bacteria are also stained with the DAPI, has a healthy nucleus. An uninfected cell without TRAIL treatment appears on the right. Bottom: Phase contrast view of the cells. The infected cell, in which the bacteria are visible in the cytoplasm, has a healthy, round appearance while the uninfected cells are apoptotic. Images are representative of three repeated experiments, and cell counts of at least 300 cells demonstrated that infected cells consistently appeared healthy (data not shown).

Surprisingly, we found multiple upregulated genes that are responsible for inhibiting apoptosis via the extrinsic pathway (Table 1 and Figure 4). This result implies that some of the apoptotic signals that occur during infection activate the extrinsic pathway of apoptosis. Signals that activate this pathway include tumor necrosis factor-α (TNF-α) and Fas ligand [13,14]. TNF-α-induced protein 8 (*TNFAIP8*) was induced in infected cells and can inhibit the TNF-α activation of caspase-8 [15]. *TNFAIP3*, *FAIM3*, and *CFLAR/c-FLIP *inhibit caspase-8 activation [16-18] and all had significant induction in infected cells. Interestingly, *TNFAIP3*, also known as *A20*, was also induced in a previous microarray analysis of *Shigella*-infected cells [4]. Based on the array results, we examined the ability of the *Shigella *to inhibit the extrinsic pathway of apoptosis (Figure 5). Using TNF-α-related apoptosis inducing ligand (TRAIL), which functions like TNF-α in the apoptosis assay, infected cells were able to inhibit apoptosis induction as seen upon nuclear staining. Therefore, the upregulation of genes required for the inhibition of the extrinsic pathway of apoptosis may be an important aspect for *S. flexneri *to inhibit apoptosis *in vivo*.

**Table 1:** A selection of upregulated genes in wildtype-infected cells compared to uninfected cells.

Gene	Function	Fold Induction
Association with the inhibition of the extrinsic pathway of apoptosis		
TNFAIP3	aka A20; inhibits TNF-alpha-induced apoptosis by inhibiting caspase-8 cleavage	9.3
TNFRSF12A	functions, in part, through the NF-κB pathway to up-regulate BCL-XL and BCL-W expression for malignant cell survival	5.1
CSE1L	binds strongly to importin-alpha; highly expressed in tumor cell lines and may play a role in inhibiting TNF-mediated cell death	3.1
FAIM3	Fas apoptotic inhibitory molecule 3; inhibits caspase-8 processing	2.7
BAG4	associates with TNFR1 and death receptor-3 to negatively regulate downstream death signaling	2.6
CFLAR	CASP8 and FADD-like apoptosis regulator; aka c-FLIP; interacts with FADD and FLICE; inhibits apoptosis via human death receptors	2.5
FAIM	Fas apoptotic inhibitory molecule; pro-survival; mediates Fas resistance produced by surface Ig engagement in B cells	2.1
PEA15	a death effector domain (DED)-containing protein; inhibits both TNFRSF6 and TNFRSF1A-mediated CASP8 activity & apoptosis	2.1
TNFAIP8	tumor necrosis factor, alpha-induced protein 8; inhibits the activated form of caspase-8	2.1
		
Interaction with caspases (IAP genes)		
BIRC4	aka XIAP; inhibits apoptosis through binding to TRAF1 and TRAF2; also inhibits caspase-3 and caspase-7	3.5
BIRC1	aka NAIP; homology to two baculoviral IAPs; suppresses apoptosis induced by various signals; binds caspase-9	3.2
BIRC5	aka survivin; with hepatitis B X-interacting protein (HBXIP) binds caspase-9; variant DeltaEx3 binds caspase-3 with BCL-2	2.8
BIRC7	aka livin; shown to interact with caspase-3, -7, & -9; degrades Smac/DIABLO	2.8

Other important genes induced in infected cells are members of the inhibitor of apoptosis (IAP) family. Expression of several IAP genes was significantly induced (Table 1). IAP upregulation has previously been observed in *Shigella-*infected cells using whole genome arrays [4]. The IAP family directly inhibits caspases [19], and caspase-3 activation is inhibited in *Shigella*-infected cells in the presence of STS [2]. The IAPs may be directly involved in preventing caspase-3 activation in infected cells treated with STS. On the other hand, if the IAP proteins are not directly involved in inhibition of caspase-3 activation in infected cells in the presence of STS, the induction of these genes could still be important to enhance the pro-survival state of the infected cell. An example is the ability of cIAP-1 to inhibit TRAF2 in TNF-α-induced apoptosis [19].

Several genes that encode proteins that associate with the mitochondrial membrane were induced in WT infected cells. For example, *BCL2 *was induced and is important for preventing permeabilization of the mitochondrial membrane [20]. Thus, the bacteria may utilize T3SS effector proteins to increase *BCL2 *expression to protect the mitochondria (Figure 4). Of note, the induction of *BCL2 *does not overcome the effects of STS since cytochrome *c *release is observed in infected cells treated with STS (see below and [2]). However, STS is a strong apoptosis inducer, and the induction of *BCL2 *in infected cells may be sufficient to prevent cytochrome *c *release in the absence of STS. The bacteria may encode T3SS effector proteins that target the mitochondria or pro-survival proteins, like BCL-2. These potential T3SS effectors would not be able to overcome the effects of STS, but would act as accessory proteins to enhance the pro-survival state of the cell. The increased expression of *BCL2 *and other genes important for protecting the mitochondrial membrane may also be a result of other pro-survival effects (see below). Interestingly, *BECN1 *expression was also induced and BECN1 has been shown to interact with BCL-2 in viral infected cells, leading to apoptosis protection [21]. Therefore, the increased expression of *BCL2 *and *BECN1 *could promote protection of *Shigella*-infected cells from apoptosis.

Additional genes that were induced in infected cells include genes important for DNA replication and repair, and cell cycle progression (Additional file 2, Table S2). *XRCC4*, *XRCC5*, *ERCC2, RAD17*, and *RAD51*, which are all key genes in DNA replication and repair [22-25], were induced. DNA damage is a signal for apoptosis [26] and maintenance of DNA integrity is an important aspect in the inhibition of apoptosis. In addition to these genes, there were also alterations in expression of genes involved in cell cycle progression or arrest in WT infected cells. One of the few repressed genes was *SPATA4*, which may be important for the S/G2 transition [27]. However, *CUL2 *and *PPP2R1B *were induced, and both promote cell cycle arrest [28-31]. Other genes important for cell cycle progression, including *E2F3 *and *TFDP2*, are induced [32,33]. As mentioned above, E2F transcription factors are regulated by JUN. The surprising changes in expression in genes that both promote and prevent cell cycle progression may reflect a complex interplay between the eukaryotic cell and the bacteria. A recent report demonstrated that the *Shigella *effector IpaB interacts with Mad2L2, leading to cell cycle arrest [34]. The authors speculate that since intestinal epithelial cells undergo rapid cell turnover, the bacteria promote cell cycle arrest to maintain infection; infected cells under cell cycle arrest resist apoptosis induction, since the cells are TUNEL-negative [34]. These results validate our observations that *S. flexneri *inhibits apoptosis. Conversely, cell cycle arrest can lead to apoptosis especially in the absence of the retinoblastoma tumor suppressor protein (pRb) [35]. Our lab has recently reported that the *S. flexneri *effectors OspF and OspB interact with pRb to modulate the immune response [36]. This interaction could also protect pRb from degradation, which would allow cell cycle arrest without leading to apoptosis. The attempt to arrest the cell cycle and the potential protection of pRb enable the bacteria to exploit cell cycle arrest and prevent apoptosis at the same time.

Finally, various genes were induced that correlate with prior observations in *S. flexneri *infection. First, *ELMO1 *was induced in infected cells. The *Shigella *effector IpgB1 binds to ELMO1 to stimulate Rac1 activity, which induces membrane ruffling during invasion of epithelial cells. Therefore, IpgB1 acts as a molecular mimic of RhoG [37], and the induction of *ELMO1 *is most likely a result of the invasion process by the bacteria. Next, the *S. flexneri *effectors IpgB2 and OspB are important for nuclear factor-kappa B (NF-κB) activation in infected cells [37,38]. The genes encoding NF-κB and proteins required for NF-κB activation were induced in infected cells (Additional file 2, Table S2 and Figure 4), including *NFKB2*. NF-κB activation is important for inducing the expression of pro-survival proteins such as TNFAIP8 [15], TNFAIP3 [16], CFLAR [18], and IAPs [39,40], which are induced in infected cells as mentioned above. In addition, *CARD15*, also known as *NOD2*, was upregulated in infected cells. Nod2 recognizes muramyl dipeptide of peptidoglycan from intracellular pathogens and activates NF-κB [41,42]. Even more important, Nod2 is also involved in the activation of the JNK pathway [43], which can lead to JUN activation. Therefore, NF-κB is a significant host factor involved in inducing a pro-survival state in the infected cell. Lastly, escape from autophagy is an important aspect of *S. flexneri *infection. Atg5 binds the bacterial protein VirG/IcsA and would normally induce autophagy; however, the bacterial protein IcsB blocks Atg5 from binding VirG/IcsA [44]. While there was an induction of *ATG12*, there was no subsequent induction in *ATG5 *or any other gene important for autophagy. This result most likely reflects the ability of the bacteria to escape autophagy. Autophagy inhibition and apoptosis inhibition may be connected [45]. Therefore, the blockage of autophagy in infected cells is likely critical for *Shigella *to survive inside epithelial cells.

In summary, *Shigella*-infected cells are in a pro-survival state compared to uninfected cells, and a major contributing factor to this state most likely was the induction of *JUN*. Genes important for blocking the extrinsic pathway of apoptosis were also induced, in addition to the IAPs, DNA repair enzymes, and genes important for NF-κB activation. Moreover, the changes in gene expression seen in infected cells can be correlated to known effects of various T3SS effector proteins. The pro-survival state of infected cells may be required for apoptosis inhibition in infected cells in the presence of strong inducers like STS. At the least, the induction of these pro-survival genes may facilitate a T3SS effector protein to directly inhibit caspase-3 activation in the presence of STS.

### *Shigella*-infected HeLa cells treated with STS compared to uninfected HeLa cells treated with STS

**Figure 6:**
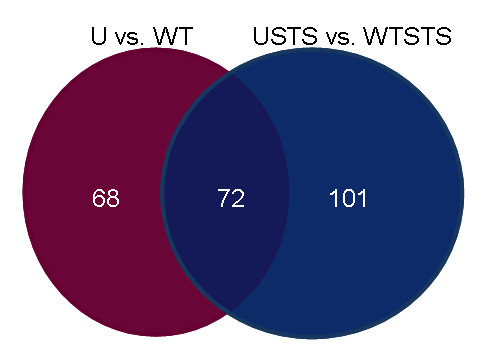
**Venn diagram comparing Uninfected versus Wildtype-infected to Uninfected cells treated with staurosporine versus Wildtype-infected cells treated with staurosporine**. There is a total of 140 differentially expressed genes in uninfected cells (U) compared to wildtype-infected cells (WT). Of those 140 genes, the same 72 genes were differentially expressed in uninfected cells treated with STS (USTS) compared to wildtype-infected cells treated with STS (WTSTS). The USTS vs. WTSTS comparison has a total of 173 differentially expressed genes.

There were 167 induced and 6 repressed genes in wildtype-infected cells treated with STS (WTSTS) compared to uninfected cells treated with STS (USTS). Among these, there were 72 genes that show the same differential expression, either induced or repressed, as in the U versus WT comparison (Figure 6). However, some of the levels of expression do vary. For example, *JUN *was induced even more under WTSTS conditions; a 27-fold induction was observed. This increase in induction compared to WT cells is most likely a direct result of the addition of STS to the infected cells. Interestingly, *FOSL2 *or *FRA-2 *was induced here but not in the U versus WT comparison. FOSL2 is a part of the AP-1 transcription factor complex and has been shown to dimerize with JUN [46]. Moreover, *LTBP3 *was induced here but not in the U versus WT comparison. LTBP3 is important in the activation of transforming growth factor-β (TGF-β) and has an AP-1 binding site in the promoter region [47]. TGF-β can lead to cell cycle arrest, but it has also been shown to be important in tumor progression [48,49]. Thus, the increase in magnitude of *JUN *expression seen in WTSTS could lead to the induction of *FOSL2 *and *LTBP3 *seen in these cells.

Another example of increased expression due to the STS was seen in the induction of *NFKBIA*; there was approximately a 7-fold increase in WTSTS cells compared to a 3-fold increase in the U versus WT comparison. *NFKBIA *is an inhibitor of NF-κB since it helps to trap NF-κB in the cytoplasm [49]. The induction of *NFKBIA *might be a response to the increased levels of NF-κB due to infection, or might be a direct effect of STS. However, the induction of this gene does not affect the expression of *NFKB2 *in WTSTS cells since *NFKB2 *has about the same level of expression as WT cells in the U versus WT comparison.

Remarkably, approximately the same anti-apoptosis genes are induced in conditions with STS compared to the above conditions without STS suggesting the same pro-survival state of the cell was induced upon infection regardless of the presence of STS. However, there are some key differences, given the fact that the bacteria inhibit apoptosis in the presence of the strong inducer STS. The only IAP induced was *BIRC2 *(Additional file 2, Table S2). *BIRC2 *encodes cIAP1, which is involved in inhibiting caspase-8 activation [39]. Interestingly, there was no induction of *BIRC4/XIAP*. Since *BIRC4/XIAP *inhibits caspase-3 activation [19], utilization of this IAP may not be important for the bacteria to inhibit caspase-3 activation in the presence of STS. Additionally, it is unlikely that the bacteria are using the XIAP already made without needing further significant production of the protein in the presence of STS over a three-hour time period. Therefore, we hypothesize that either the bacteria use a T3SS effector protein to directly inhibit caspase-3 activation in the presence of STS, or that the bacteria indirectly block caspase-3 activation by upregulation of other pro-survival genes. For example, genes required for NF-κB activation were again induced in WTSTS cells (Additional file 2, Table S2), leading to the same pro-survival effects outlined above. Interestingly, *TRAF2 *was induced in WTSTS, and this induction was not seen in the U versus WT comparison. TRAF2 is important for caspase-8 activation and is induced by NF-κB [39]. In addition, TRAF2 can activate the JNK pathway via MEKK1 leading to JUN induction [50]. Also in support of protection through NF-κB, *IER3 *was induced in WTSTS cells compared to USTS cells. IER3, also known as IEX-1L, is involved in protecting cells from TNF-induced apoptosis, and IER3 is regulated by NF-κB [51]. Additional possibilities for the inhibition of caspase-3 in the presence of STS include the repression of pro-apoptotic pathways (see below). Once we identify the bacterial protein required for apoptosis inhibition, we can investigate how this protein functions in the eukaryotic cell.

There were two genes that appear in both sets of comparisons but show opposite directions of expression. First, *NALP1 *was induced in U versus WT but repressed in USTS versus WTSTS. NALP1 is part of the inflammasome in which pro-inflammatory caspase-1 activation leads to interleukin-1β (IL-1β) processing, especially in the presence of LPS [52]. NALP1 is suppressed by BCL-2 and BCL-X_L _to reduce caspase-1 activation and IL-1β production [53]. *BCL2 *was induced in WTSTS cells (see below). The *Shigella *effector IpaB binds and activates caspase-1 in macrophages, leading to IL-1β secretion and cell death via pyroptosis [1]. There have not been any studies regarding IpaB and caspase-1 activation in epithelial cells. While it may not be the primary method of apoptosis inhibition, NALP1 repression or inhibition of NALP1 by BCL-2 may be an important mechanism for the pro-survival state of the infected epithelial cell in the presence of STS. This finding may be a crucial explanation for the differences in bacterial-induced cell death in macrophages and bacterial-induced cell survival in the epithelial cells. Second, *EDARADD *was repressed in U versus WT while it was induced in USTS versus WTSTS. EDARADD acts as an adaptor protein for EDAR to recruit TRAF2 proteins during NF-κB activation [54,55]. This induction of *EDARADD *possibly enhances the pro-survival effect of NF-κB activation in the presence of STS as described above.

The comparison between USTS to WTSTS treatments revealed numerous differences in p53-associated genes and pRb-associated genes that were not seen in the U versus WT comparison (Table 2). p53 is a transcription factor and tumor suppressor, and can induce apoptosis by activating various targets that lead to mitochondrial permeabilization [56,57]. p53 itself was not altered in USTS versus WTSTS, and JUN is known to be a direct repressor of p53 [7]. Therefore, the induction of *JUN *most likely had a significant effect on the expression of p53 in infected cells. However, *TP73L *or *TP63*, a homolog to p53 that can induce apoptosis by activating pro-apoptotic genes including *BAX*, *APAF1*, and *caspase-9 *[57], had increased expression in WTSTS cells. The induction of *TP63 *most likely lead to the increased expression of *BAX*, *APAF1*, and *caspase-9 *seen in WTSTS cells. However, increased levels of these proteins and the subsequent activation of the proteins by STS had no effect on WTSTS cells since *S. flexneri *inhibits apoptosis after caspase-9 activation [2]. Interestingly, TP63 can also induce *caspase-8 *and *caspase-3 *[57], but these genes were not induced in WTSTS cells. Finally, many genes, in which the gene products affect p53, were upregulated. For example, *TP53BP2 *was induced in WTSTS cells. TP53BP2 is a part of the apoptosis-stimulating protein of p53 (ASPP) family of p53-interacting proteins and enhances p53 binding to DNA for transcriptional activation of pro-apoptotic genes [58]. Also, *PPP2CA*, which was induced in WTSTS cells, induces the expression of p53 and can lead to G2/M cell cycle arrest [59]. *P53AIP1 *was induced in WTSTS cells compared to USTS cells, and is a p53-dependent gene whose gene product binds BCL-2 to cause cytochrome *c *release from the mitochondria [60]. Due to the mitochondrial permeabilization of *Shigella*-infected cells in the presence of STS, it is not surprising that these p53-regulated genes were induced. Despite the induction of p53-responsive genes, *p53 *itself was not induced in WTSTS or in WT cells most likely due to significant *JUN *induction since JUN represses p53.

**Table 2:** A selection of upregulated genes in wildtype-infected cells with staurosporine compared to uninfected cells with staurosporine.

Gene	Function	Fold Induction
Association with p53		
TP53BP2	a member of the ASPP (apoptosis-stimulating protein of p53) family of p53 interacting proteins	3.3
TP73L	aka tumor protein p63; homolog to p53	3.1
JUND	member of the JUN family; a component of the AP1 TF complex; proposed to protect cells from p53-dependent senescence and apoptosis	2.8
P53AIP1	P53-regulated apoptosis-inducing protein 1; p53AIP1 regulates the mitochondrial apoptotic pathway	2.7
CUL4A	functions as an E3 ligase & with MDM2, contributes to p53 degradation; assists in nucleotide excision repair in a complex with DDB	2.6
LRDD	interacts FADD & MADD; may function as an adaptor protein in cell death-related signaling processes; a p53 effector (induces apoptosis)	2.5
NEDD8	similar to ubiquitin; conjugated to p53 via MDM2, thereby inhibiting the transcriptional activity of p53	2.2
PPP2CA	the phosphatase 2A catalytic subunit; inhibits cell growth and activates p53 & p21 (p53-responsive); overexpression leads to G2/M arrest	2.2
MDM4	contains a RING finger domain & a putative nuclear localization signal; interacts with p53 (stabilizes to counteract MDM2)	2.0
		
Association with pRB		
RBBP4	involved in chromatin remodeling & transcriptional repression associated with histone deacetylation; binds directly to pRB	3.7
JARID1A	aka RBBP2; binds with pRB for tumor suppressive functions; may interact with rhombotin-2	2.7
RBBP5	a ubiquitously expressed nuclear protein; binds pRB, which regulates cell proliferation	2.3
RBBP6	binds to unphosphorylated but not phosphorylated pRB; also binds p53; blocks suppression of adenoviral E1A protein	2.0

There were also induced genes in WTSTS cells that are responsible for suppressing p53 in addition to *JUN*. These genes include *JUND *[61], *CUL4A *[62], and *NEDD8 *[63] (Additional file 2, Table S2). JunD, which is in the AP-1 transcription factor complex like JUN, is also important for inhibiting TNF-stimulated apoptosis. JNK increases the expression of JunD, and JunD acts with NF-κB to increase the expression of *cIAP2 *[64]. *GADD45 *(or *GADD45A*) which is a p53-responsive gene that recognizes damaged chromatin and facilitates topoisomerase cleavage activity to cause DNA damage [65], was induced approximately 10-fold in WTSTS cells. In addition, *GADD45A *expression may be regulated by AP-1 complexes containing JunD [66]. This induction may be a result of the high induction levels of the genes associated with AP-1 complexes, namely *JUN, JUND*, and *FOSL2*. The pro-apoptotic phenotype of *GADD45A *does not affect WTSTS cells. Another gene that is responsible for suppressing p53 and directly degrades the protein, *MDM2 *[67], was induced in WTSTS cells. The induction of genes important for enhancing and suppressing p53 most likely represents an attempt by the cell to undergo apoptosis in the presence of STS, especially since p53 enhances cytochrome *c *release from the mitochondria [56]. Conversely, the bacteria are inhibiting apoptosis and inducing a pro-survival state of the cell, which most likely explains the induced expression of genes responsible for suppressing p53. Interestingly, *MDM2 *was induced in the U versus WT comparison in addition to the significant induction of *JUN*. The bacteria may directly upregulate *MDM2 *expression or the upregulation could be a response of the eukaryotic cell to the pro-survival state seen.

pRb-associated genes occur more in the USTS versus WTSTS comparison than in the U versus WT comparison. The *RBBP4, RBBP5/RBQ-3, RBBP6*, and *JARID1A *or *RBBP2 *genes were all induced in WTSTS cells and are important for pRb function [68-71]. In fact, *RBBP4 *is repressed in cervical cancer due to human papillomavirus infection [69]. *RBBP6 *has been shown to bind p53, inhibit adenoviral E1A from binding pRb, and may have a ubiquitin-like domain [72]. In addition, *SERPINB2*, which represses pRb pro-apoptotic signal transduction [73], was induced in WTSTS cells. As mentioned above, the bacteria may require pRb function to prevent apoptosis while attempting to cause cell cycle arrest. In support of this hypothesis, there were even more genes induced in the USTS versus WTSTS comparison that are involved in cell cycle arrest or progression than the genes induced in the U versus WT comparison. For example, *CUL1*, *CUL3, APPBP1*, and *ESPL1/ESP1 *are induced. These genes are important for regulation of the cell cycle [74-77]. The observations further highlight the interplay between the bacteria attempting to arrest the cell cycle while the eukaryotic cell attempts to progress the cell cycle as described above.

Finally, there were more induced genes whose gene products are localized to the mitochondria in STS conditions (Additional file 2, Table S2). For example, *DIABLO *and *HTRA2 *were induced in WTSTS cells and encode proteins responsible for inhibiting IAPs upon release from the mitochondria [78]. The pro-apoptotic *BAX, BCL2L11, BID, BNIP3L*, and *BOK *proteins [78] were all induced in WTSTS cells as well. These pro-apoptotic genes do not affect the inhibition of caspase-3 by *Shigella*, especially since cytochrome *c *release occurs in the presence of STS in infected cells [2]. Finally, the pro-survival *BCL2L2 *or BCL-*W *and *GLRX2 *[78,79] genes, in addition to the induction of *BCL2 *and *CYCS *(encodes cytochrome *c*) that also occurs in WT cells, were induced in WTSTS cells. *GLRX2 *encodes glutaredoxin, which protects the mitochondria from oxidative stress [79]. The induction of these pro-survival genes most likely reflects an attempt of the infected cell to repair or maintain mitochondrial integrity during STS treatment of infected cells.

In summary, the changes in gene expression in the USTS versus WTSTS comparison were similar to the changes seen in the U versus WT comparison. Nevertheless, there were some key distinctions that include higher levels of induction of some genes, opposite expression of genes in the presence of STS, enhancement of the pro-survival state related to NF-κB, induction of genes related to p53 and pRb, and the induction of more genes associated with the mitochondrial membrane. The majority of these changes most likely represent the pro-survival state induced by *Shigella*, and these changes were enhanced upon STS exposure. However, a few changes, including the repression of *NALP1*, may have a direct role in apoptosis inhibition by *Shigella*.

### *Shigella*-infected HeLa cells treated with STS compared to *Shigella*-infected HeLa cells

The purpose of this comparison was to measure the changes in infected cells that are required for apoptosis inhibition in the presence of STS. Surprisingly, the SAM analysis revealed no significant genes. When the less stringent student's t-test was used to analyze the data, we did find changes in gene expression (Additional file 2, Table S2); however, the fold changes were not as high as the other comparisons. Approximately 80% of the changes were less than two-fold and the highest induction or repression was approximately 3-fold. Thus, the array results demonstrated that there were few significant differences between the two conditions and suggested that the bacteria induce the same pro-survival state in infected cells regardless of the presence or absence of STS. Therefore, STS has no overall significant effect on infected cells. The cluster diagram in Figure 3 supports this hypothesis since WT cells and WTSTS cells are interspersed while U cells cluster away from USTS cells. Also in support of this hypothesis, there was no change in expression of *JUN*, *BIRC2/cIAP1, TRAF2*, or *NFKB2*. The absence of changes in these key genes indicates that *Shigella *infection itself has a pro-survival effect on the eukaryotic cell that is not altered by the presence of STS.

Of the few additional changes seen, it is interesting to see that *CASP10 *was repressed in WTSTS cells compared to WT cells. Caspase-10 is activated in the extrinsic pathway of apoptosis, and etoposide, a chemotherapeutic agent and cytotoxic drug, induces *CASP10 *expression in a p53-dependent manner [80,81]. In addition, caspase-10 is activated after cytochrome *c *release to amplify caspase-9 and caspase-3 activation in the presence of etoposide. In fact, caspase-10 activation can be inhibited if cytochrome *c *release is inhibited. More importantly, a dominant-negative form of caspase-10 is able to inhibit the activation of caspase-3 in the presence of etoposide [81]. Since *S. flexneri *inhibits caspase-3 activation despite cytochrome *c *release in the presence of STS, caspase-10 could be a eukaryotic target for the bacteria to utilize to interfere with caspase-3 activation and inhibit apoptosis. Alternatively, p53 inhibition could reduce caspase-10 levels, which demonstrates the importance of the inhibition of p53 activity that occurs during infection.

Finally, it is important to note that *IKBKG/NEMO *was induced in the WTSTS cells compared to WT cells (Additional file 2, Table S2). NEMO is the regulatory subunit of the IκB kinase (IKK) complex that, when activated, phosphorylates the IκB proteins. Phosphorylation leads to ubiquitination of IκB proteins, thereby releasing NF-κB and allowing NF-κB to enter the nucleus for transcriptional activation. In addition, cIAP1 ubiquitinates NEMO in response to TNF-α stimulation, which is required for NF-κB activation [82]. The upregulation of *NEMO *is most likely the result of continued NF-κB activation in *Shigella*-infected cells in the presence of STS. Nevertheless, this comparison highlights the fact that the infected cell is under the same pro-survival state regardless of the presence or absence of STS. This strong apoptosis inducer, therefore, has little overall effect on the infected cells.

### *In situ *hybridization analysis to confirm the microarray results

**Figure 7:**
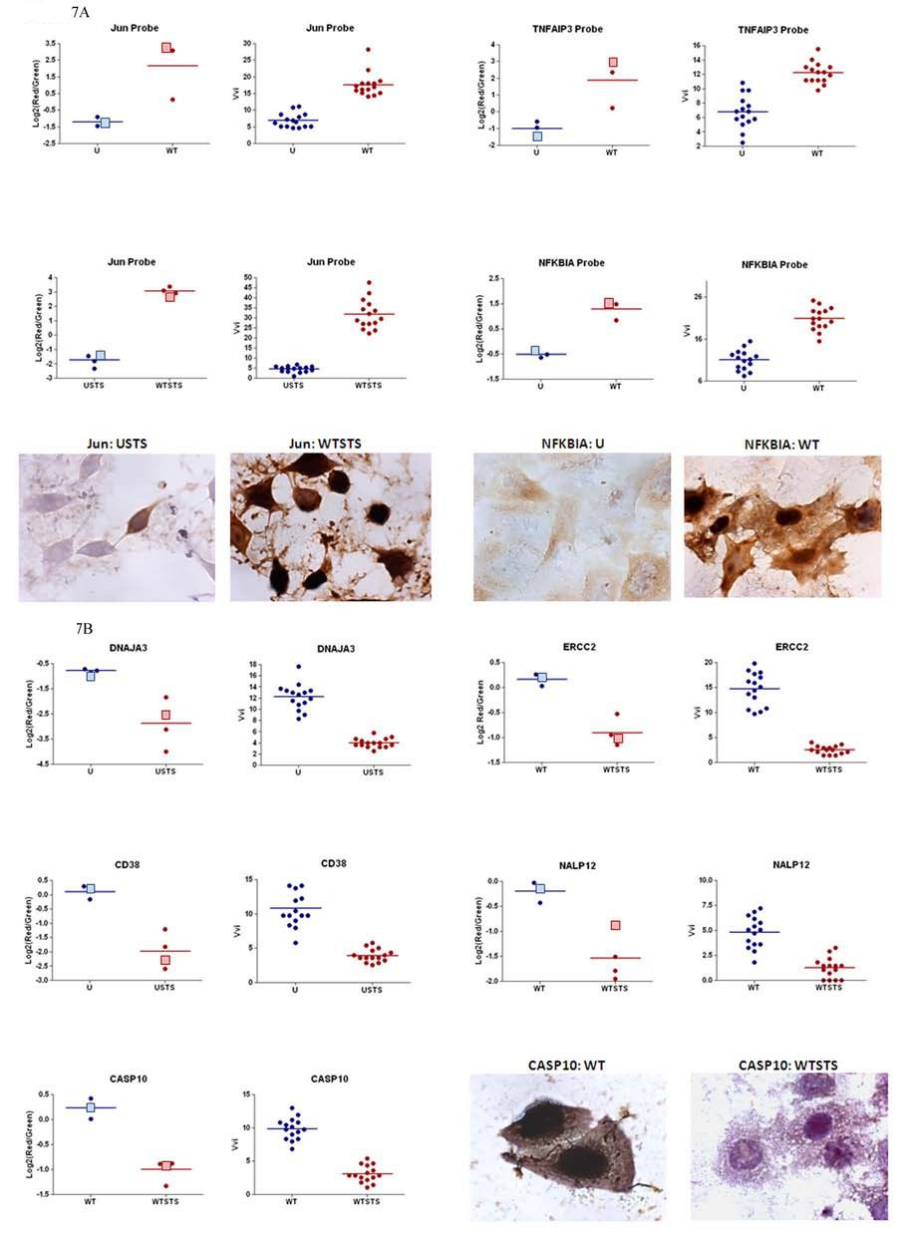
**Comparison of the microarray and *in situ *hybridization results**. The log_2 _of the red/green ratio for the microarray analysis is plotted on the left for the specified probe while the volumetric density (Vvi) for the *in situ *hybridization (ISH) analysis is plotted on the right for the specified probe. For the microarray analysis, the data for each time point of treatment (refer to Figure 2) are plotted with a line representing the mean of the data. The bigger, light-colored squares represent the time points that correlate with the ISH data. For the ISH, the Vvi for 15 fields at 400× magnification for the single time point is plotted with a line representing the mean of the data. A student's t-test was performed on the ISH data, and all p-Values were < 0.0001, indicating that there is a significant difference between uninfected cells and infected cells for the expression of each gene tested. The images represent some of the ISH reactions for the specified treatment condition and probe. Images were taken at 1000× magnification. U = uninfected cells, WT = infected cells, USTS = uninfected cells treated with staurosporine, and WTSTS = infected cells treated with STS. A: Induced genes. B: Repressed genes.

We used *in situ *hybridization (ISH) analysis as previously described [83] to quantify the mRNA expression of several genes and to confirm the results of the microarray analysis. ISH has been shown to be as sensitive as real-time RT-PCR [84] and is therefore an appropriate method to confirm the microarray results. Biologically independent samples were collected and analyzed with biotin-labeled probes representing genes that showed significant fold changes in the microarray results, namely *JUN, TNFAIP3, NFKBIA, CASP10, NALP12, ERCC2, DNAJA3*, and *CD38*. As shown in Figure 7A, each probe showed the same trend for the ISH analysis as was seen in the microarray analysis, namely a significant increase in expression of each gene in cells infected with bacteria as indicated by a positive, brown staining reaction. For the *JUN *probe, the same result was seen regardless of the presence of STS. Repressed genes also had the same trend for the ISH analysis as was seen in the microarray analysis (Figure 7B). All reactions utilized a control in which PBS was added in place of the probe. Additional controls included DNase, RNase, or DNase plus RNase treatment of the samples prior to the addition of the probes. These treatments degrade the targets in the tissue while allowing the probe to be applied to the sample, which ensures that the brown reaction does not result from nonspecific binding of the biotin-labeled probe. All controls were negative for the peroxidase reaction (data not shown). Therefore, the ISH analysis validates the microarray results reported above.

## Conclusion

The identification of the *Shigella *proteins required for the inhibition of apoptosis and the mechanism by which the proteins inhibit apoptosis will help define which changes in eukaryotic gene expression are relevant for STS inhibition. However, the changes in eukaryotic gene expression described here appear to be important for enhancing the pro-survival state of the infected cell in the absence of a strong apoptosis inducer like STS. Future studies will define the importance of the induction of certain genes. For example, siRNA studies to knock down JUN, the IAPs, or NF-κB expression will help to determine which changes are required for apoptosis inhibition upon infection. In addition, analysis of the extrinsic pathway of apoptosis will allow us to determine if inhibition occurs prior to caspase-8 or caspase-3 activation, as well as identify which proteins in Table 1 are involved. The alterations in eukaryotic gene expression reported here are important to fully understand how *Shigella *inhibits apoptosis in epithelial cells.

There are other bacterial pathogens that inhibit apoptosis [85] and some of these pathogens have been utilized in similar microarray analyses to identify changes in eukaryotic gene expression in infected cells. Studies with *Neisseria gonorrhoeae*, which can inhibit STS-induced apoptosis at the mitochondrial level, found two to eight-fold upregulation of *BFL-1, COX-2, MCL-1*, and *cIAP2 *in infected cells [86]. *Mycobacterium tuberculosis *is able to induce cell death in alveolar macrophages while it can prevent apoptosis in alveolar epithelial cells. *M. tuberculosis *infection of epithelial cells results in increased expression of *BCL2 *and *pRb*, decreased expression of *BAX *and *BAD*, and no change in *p53 *expression despite a large increase in expression of *p53 *in infected macrophages. In addition, the macrophages show significant inhibition of *pRb *[87]. The *p53 *and *pRb *observations are similar to the changes we report in *S. flexneri *infection of epithelial cells, both in the presence and absence of STS. Another similarity to *Shigella *infection is seen with the pathogen *Edwardsiella tarda*, which upregulates NF-κB target genes, including *cIAP2 *and *TRAF1 *in macrophages [88]. Finally, analysis of *Rickettsia rickettsii *infected endothelial cells in the presence of STS revealed induced expression of *TRAFs*, many genes the products of which localize to the mitochondria, several *IAPs, AKT1*, and *p53 *[89]. Like the above pathogens, *S. flexneri *induces similar changes in eukaryotic gene expression in order to inhibit apoptosis. However, each pathogen is unique in that it may employ a different method to inhibit apoptosis. For example, while *S. flexneri *inhibits capsase-3 activation, other pathogens like *N. gonorrhoeae *prevent mitochondrial permeabilization. Despite these differences, a common theme has emerged in that the bacteria induce a pro-survival state in infected cells, which results in similar changes in eukaryotic gene expression.

Knowing that *S. flexneri *inhibits STS-induced apoptosis at the level of caspase-3 activation [2] and given the changes in eukaryotic gene expression and resistance to TRAIL-induced apoptosis reported here, we propose that *S. flexneri *blocks apoptosis at multiple checkpoints in infected cells (Figure 4). Upon infection of epithelial cells, the bacteria either directly induce protection of the mitochondria by secreting T3SS effector proteins or indirectly protect the mitochondria by upregulating several eukaryotic genes including *JUN*, *NFKB2*, and *BCL2*. This possibility is supported by the evidence that there is no cytochrome *c *release upon normal infection with *Shigella *[2]. Another level of protection induced upon infection is resistance to inducers of the extrinsic pathway of apoptosis, such as TRAIL. Upregulation of *TNFAIP3, TNFAIP8, TNFRSF12A, FAIM3*, and *CFLAR *are important to inhibit caspase-8 activation, and may be direct targets of *Shigella *T3SS effector proteins or result from NF-κB activation. It is important to inhibit apoptosis from the extrinsic pathway since many *in vivo *stimuli are present during infection such as TNF-α and Fas ligand [13,14]. Finally, the bacteria provide downstream protection and directly inhibit caspase-3 activation to prevent apoptosis, which is only evident when strong apoptosis inducers like STS are used. This downstream block provides protection if the invading *Shigella *fail to inhibit apoptosis at upstream checkpoints. While STS can overcome many of the pro-survival effects like protection of the mitochondria, the chemical cannot overcome the protection of caspase-3 cleavage induced by the bacteria. In addition, the upregulation of genes to suppress the effects of p53 enhance the pro-survival effects of the infected cell challenged with apoptosis inducers. Future experiments will determine which bacterial T3SS effector protein(s) and which eukaryotic genes are required for *S. flexneri *to inhibit apoptosis. The evidence presented here clearly shows that there are multiple steps required for *Shigella *to successfully prevent apoptosis in infected epithelial cells. Without this protection, *Shigella *would not have an efficient means of survival *in vivo*.

## Methods

### Bacterial strains used and growth conditions

The strain used in the study was the wildtype *S. flexneri *serotype 2a strain 2457T. Bacteria were routinely cultured at 37°C either in Luria-Bertani broth (LB) with aeration or on tryptic soy broth plates with 1.5% agar and 0.025% Congo red (Sigma).

### Immunofluorescence analysis

After the varying STS exposures on the uninfected cells (Figure 1), cells were fixed with 3% formaldehyde (36% stock; Sigma) and 0.2% glutaraldehyde (25% stock; Sigma) in 1× PBS for 5 minutes at 4°C. Immunofluorescence analysis was performed as previously described [2]. For Bad staining, a rabbit anti-Bad antibody (Cell Signaling Technology) was used in conjunction with a goat anti-rabbit immunoglobulin G (IgG) antibody conjugated to Alexa-594 (Invitrogen). An additional antibody that recognizes the phosphorylated form of Bad (phospho-Bad (Ser112), Cell Signal Technology) was also used with a goat anti-mouse IgG antibody conjugated to Alexa-594 (Invitrogen). For the cytochrome *c *release staining, the staining procedures were followed as described in the protocol provided by Molecular Probes. For the activated caspase-3 staining, a primary anti-human cleaved caspase-3 antibody (Cell Signaling Technology) was used with the same goat anti-rabbit secondary antibody above. To visualize nuclei, 5 mg/ml of 4,6-diamido- 2-phenylindole (DAPI; Molecular Probes) was diluted 1:1,000 in 1× phosphate buffered saline (PBS) and added to the monolayers for 20 min at room temperature in the dark. For all immunofluorescence experiments, antifade reagent (Molecular Probes) was added before coverslips were applied after the staining procedure. Samples were stored in the dark at 4°C and analyzed with an Olympus BX60 fluorescence microscope with an attached digital camera using ×100 magnification.

### Apoptosis assay and RNA isolation

The apoptosis assay was performed in HeLa cells as previously described in which infections occurred at a multiplicity of infection of 100 bacteria per HeLa cell [2] and the various treatment conditions are provided in Figure 2. In both the presence and absence of STS, 90 percent infection was achieved as previously demonstrated [2]. The STS exposure times were modified to reflect key points in the apoptosis pathway (see Results and Discussion). After the apoptosis assay, the monolayers were washed with 1× PBS and RNA was isolated using TRIzol reagent. RNA was extracted from the TRIzol using chloroform, precipitated using isopropyl alcohol, and cleaned using the RNeasy kit (Qiagen). DNase treatment occurred directly on the columns, and after washes, the RNA was resuspended in 30 μl RNase-free water. The reference RNA for all hybridizations consisted of a pooled sample of RNA isolated from normal, healthy HeLa cells. The RNA concentration of the treatments and the reference was quantitated by determining the OD_260 _and the RNA integrity of all the samples was analyzed on a 1% agarose gel. All RNA was pure and not degraded by the isolation procedure (data not shown).

The apoptosis assay was modified to investigate the extrinsic pathway of apoptosis, which was also performed in HeLa cells using recombinant tumor necrosis factor-α related apoptosis inducing ligand (TRAIL, Calbiochem). The apoptosis assay was performed as previously described [2]. Simply, TRAIL was substituted for STS in a three-hour incubation at a concentration of 3.4 μg/ml.

### Microarray hybridization and analysis

The treatment RNA and reference RNA were concentrated to 5 μg RNA in 12 μl RNase-free water for cDNA synthesis. The FairPlay III kit (Stratagene) was used for preparing labeled cDNA with some modifications. 500 ng/μl random hexamer solution was used in the reaction, and once the cDNA was synthesized, the treatments and reference were purified using ethanol precipitation in which the samples were placed at -20°C for 1 hour. Next, the NHS-Ester containing dye coupling reaction was performed according to the protocol. The reference and treatment cDNA were subsequently indirectly labeled with Cy3 (green) and Cy5 (red) fluorophores, respectively. The samples were then purified to remove uncoupled dye, and the labeled cDNA was eluted in 50 μl of 10 mM Tris base, pH 8.5. The cDNA was analyzed via a spectrophotometer to determine dye incorporation and cDNA yield. The reference sample was mixed with each of the treatments so that each treatment had 1 μg of cDNA and 1 μg of reference cDNA. The samples were concentrated in the speed vacuum on medium heat to 44 μl, and then 11 μl of 10× blocking agent (7.5 μl 10× Agilent blocking reagent, 2 μl human COT-1 DNA (10 μg/μl), 1 μl poly d(A)_40-60 _(8 μg/μl), 1 μl yeast tRNA (25 μg/μl)) was added to the samples. The mixtures were heated to 98°C for 2 minutes, cooled briefly, and the 2× hybridization buffer was added (HI-RPM, 55 μl), resulting in a final volume of 110 μl for each sample. The samples were loaded onto the ExonHit Therapeutics microarrays (451 apoptosis-specific genes) for hybridization at 65°C. After overnight hybridization, the arrays were washed and scanned using a 4000A scanner and the GENEPIX 3.0 software.

Data were collated using the Stanford Microarray Database [90] in which spots showing obvious abnormalities were excluded from the analysis and an uncentered metric was used during the clustering. Treatment conditions across all time points were grouped into a single condition, and each condition was then compared to the other treatment conditions to reveal changes in eukaryotic gene expression that are important for apoptosis inhibition in the presence of STS in *Shigella*-infected cells. This grouping allowed us to identify changes in gene expression in only those genes showing the most consistent and significant changes within each treatment group. The significance analysis of microarray (SAM) program version 2.20 and the student's t-test with a p-value cutoff of less than 0.01 were used to generate the list of significant genes. The false discovery rate (FDR) of the four pairwise comparisons did not exceed 3.1%. The genes in Additional file 2, Table S2 were categorized by function and/or pathway using the gene descriptions provided by NCBI's Entrez Gene. Figure 3 was generated by average linkage clustering using the hierarchical clustering algorithm of the Cluster 2.11.0.0 program.

### *In situ *hybridization analysis

*In situ *hybridization (ISH) was performed as previously described [83] with some modifications to confirm the microarray results. Infections were performed in HeLa cells with wildtype bacteria for 5.5 hours in DMEM plus 50 μg/ml gentamicin. When used, STS was applied at a 4 μM concentration for the last 2.5 hours of the assay. Uninfected cells received the same treatments and incubation times as infected cells in the appropriate comparisons. These time points were chosen to see the overall effect of infection and/or STS exposure on the cells. Afterwards, the HeLa cells were fixed with 3% formaldehyde (36% stock; Sigma) and 0.2% glutaraldehyde (25% stock; Sigma) in 1× PBS overnight at 4°C. For the ISH analysis, the probes and sequences used to generate the probes are listed in Table 3. The probe sequences were designed within the sequence of the corresponding microarray probe, and anneal to the cDNA sequence of the respective gene. All sequences are 32 nucleotides in length, and these sequences were analyzed with BLAST to ensure specificity to the gene target. All probes were synthesized in the Synthesis and Sequencing Facility, Biomedical Instrumentation Center, Uniformed Services University of the Health Sciences (USUHS; Bethesda, Maryland). The 5' end of each oligonucleotide was labeled with biotin. After probe hybridization, avidin-conjugated peroxidase and 3,3'-diaminobenzidine (DAB) tablets (Sigma) were used to detect the probes, which results in a brown reaction. Finally, the samples were counterstained with hematoxylin QS (Vector) to visualize the cells and were mounted with VectaMount.

**Table 3:** Probes and sequences used for the *in situ *hybridization experiments.

Probe	Sequence
*JUN*	5' - CCTGGGTTGAAGTTGCTGAGGTTTGCGTAGAC - 3'
*TNFAIP3*	5' - TGCGCTGGCTCGATCTCAGTTGCTCTTCTGTC - 3'
*NFKBIA*	5' - TCATGGATGATGGCCAAGTGCAGGAACGAGTC - 3'
*CASP10*	5' - TTGCCAGGACTCCTGCGGTAAGGCTTCCAAGA - 3'
*NALP12*	5' - GCCACAGCTATCCAGCCACAGTTTCTGGAGTC - 3'
*ERCC2*	5' - ATCTCTGGCACAGTTCTTGAGCAGTAGATGAG - 3'
*DNAJA3*	5' - GGTAATGTTTTGTTCCTGTGAGGCTGACACCA - 3'
*CD38*	5' - CGCTGGACCTGTGTGAACTGATGGGCCAGATC - 3'

In the data analysis, mRNA expression was quantified by a Nikon Eclipse E800 microscope as a brown reaction. Fifteen random fields were counted at 400× magnification for each preparation, according to a modification of the point-counting stereological method [91] using an intraocular reticle of 27-mm diameter, covering 3578 μm^2 ^(Kr409, Klarman Rulings) [83]. Volumetric density (Vvi) analysis of the different probes was performed to measure the number of intersections of the grid that fell on the positive brown reaction. Statistical significance was determined using the student's t-test to compare treatment groups.

## Authors' contributions

CSF designed the experiments, collected the data, analyzed and interpreted the results, and drafted the manuscript. DSM assisted in the design, execution, and data analysis for the microarray experiments, and helped draft the manuscript. CS-M, AD, and AR assisted in the collection and interpretation of data for the *in situ *hybridization experiments. ATM assisted in the experimental design and critically revised the manuscript. All authors read and approved the final manuscript.

## Supplementary Material

Additional file 1 **Table S1: Data for spots that show statistically significant differences in the indicated pairwise analyses**. The log_2 _red to green ratio for all spots that showed statistically significant differences in expression in the four pairwise analyses are provided. Click here for file

Additional file 2 **Table S2: Lists of genes in all comparisons categorized by function**. On the first worksheet, there is a list of genes in all comparisons categorized by function. On the second through fifth worksheets, the list of genes from each comparison with a brief description of gene function obtained from NCBI's Entrez Gene is provided. For each comparison, induction or repression refers to the changes in gene expression in the second treatment relative to the first treatment. Each worksheet can be accessed by clicking on the tabs at the bottom of the Excel page. Click here for file
